# Progress in PRRSV Infection and Adaptive Immune Response Mechanisms

**DOI:** 10.3390/v15071442

**Published:** 2023-06-27

**Authors:** Huanchang Cai, Hewei Zhang, Huai Cheng, Min Liu, Shubo Wen, Jingqiang Ren

**Affiliations:** 1 Wenzhou Key Laboratory for Virology and Immunology, Institute of Virology, Wenzhou University, Wenzhou 325035, China; 2 College of Food and Drugs, Luoyang Polytechnic, Luoyang 471099, China; 3 Animal Diseases and Public Health Engineering Research Center of Henan Province, Luoyang 471000, China; 4 Preventive Veterinary Laboratory, College of Animal Science and Technology, Inner Mongolia Minzu University, Tongliao 028000, China

**Keywords:** PRRSV, persistent infection, adaptive immunity, non-neutralizing antibodies

## Abstract

Since its discovery, Porcine reproductive and respiratory syndrome (PRRS) has had a huge impact on the farming industry. The virus that causes PRRS is Porcine Reproductive and Respiratory Syndrome Virus (PRRSV), and because of its genetic diversity and the complexity of the immune response, the eradication of PRRS has been a challenge. To provide scientific references for PRRSV control and vaccine development, this study describes the processes of PRRSV-induced infection and escape, as well as the host adaptive immune response to PRRSV. It also discusses the relationship between PRRSV and the adaptive immune response.

## 1. Introduction

Porcine reproductive and respiratory syndrome (PRRS), which initially appeared in the United States and Europe in the late 1980s, is a highly infectious disease with high contact and fatality rates. Porcine Reproductive and Respiratory Syndrome Virus (PRRSV) was identified as the causative agent of PRRS through epidemiological analysis and Koch hypothesis studies of initial isolates from both locations [1,2]. A single-stranded, positive-stranded, non-segmented RNA virus with a capsid, PRRSV is a member of the genus *Arterivirus* and the family *Arteriviridae* of the order *Nidovirales* [3]. The interior nucleocapsid of the virus is around 25–30 nm in diameter with icosahedral symmetry, and the majority of the viral particles are spherical or oval, with a smooth appearance under the electron microscope [4]. According to genome sequencing results, PRRSV can be classified into PRRSV-I (European type, prototype strain Lelystad virus) and PRRSV-II (North American type, prototype strain VR-2332 virus) [5,6]. PRRSV-II is predominantly endemic in China, showing diverse pathogenicity, genetic variability, and periodic occurrence of severe disease phenotypes. However, in recent years, PRRSV-I has spread throughout central, northern, southern, eastern, northeastern, and southwestern China, with at least 22 provinces reporting high PRRSV-I prevalence. Epidemiological studies are now being conducted, and PRRSV-I strain surveillance is being increased [7,8,9].

Pigs are typically infected by PRRSV in their monocyte–macrophage system. Pulmonary alveolar macrophages (PAMs) and dendritic cells (DCs) are major members of the mononuclear phagocyte system that deliver antigens to T cells and release cytokines that regulate inflammation and the immunological responses that are crucial for the activation of adaptive immune responses [10,11]. PRRSV-infected hosts exhibit typical immunological characteristics, including persistent viremia, strong suppression of innate cytokines, delayed appearance of neutralizing antibodies, induction of non-neutralizing antibodies, and dysfunction of the natural killer (NK) cell population [12]. We reviewed the connection between PRRSV and the adaptive immune response and summarized PRRSV infection mechanisms, the host’s adaptive immune response to PRRSV, and PRRSV tactics to evade cellular and humoral immunity. For the convenience of readers, the following is a list of professional terms and abbreviations used in this article.

## 2. Mechanism of PRRSV Infection

The only natural hosts for PRRSV are pigs. As soon as the PRRSV glycoprotein binds to the sialic acid adhesins on porcine macrophages, clathrin-dependent endocytosis is triggered, allowing the virus to enter the cell and initiate transcription, assembly, and release. The body will respond with an immunological reaction when the virus proliferates and releases into the internal environment. However, PRRSV’s immunological escape strategy causes tissue lesions and inflammation, which have a significant negative impact on pigs of all ages. For sows in late gestation, it causes reproductive failure, which is characterized by premature stillbirths, partially autolyzed fetuses, and mummified fetuses. Infected weak newborn piglets will not survive to weaning. Moreover, PRRSV causes respiratory disease in young and growing pigs, resulting in secondary bacterial and viral infections, slow growth, and, in severe cases, death [13].

### 2.1. PRRSV Infection and Receptor Proteins

PRRSV infection has strict cell and tissue tropism [14], and in addition to PAMs, PRRSV can proliferate in many cell types, such as porcine testicular spermatogenic cells and respiratory epithelial cells, a property associated with specific receptor proteins during PRRSV infection. The main receptors identified so far include the cysteine-rich scavenger receptor (CD163), sialoadhesin (Sn or CD169), heparin sulfate (HS), vimentin, cluster of differentiation 151 (CD151), non-muscle myosin heavy chain 9 (MYH9), heat shock protein member 8 (HSPA8), T-cell immunoglobulin and mucin structural domain (TIM), and dendritic cell-specific intercellular adhesion molecule-3-grabbing non-integrin (DC-SIGN or CD209) [15,16].

**Figure 1:**
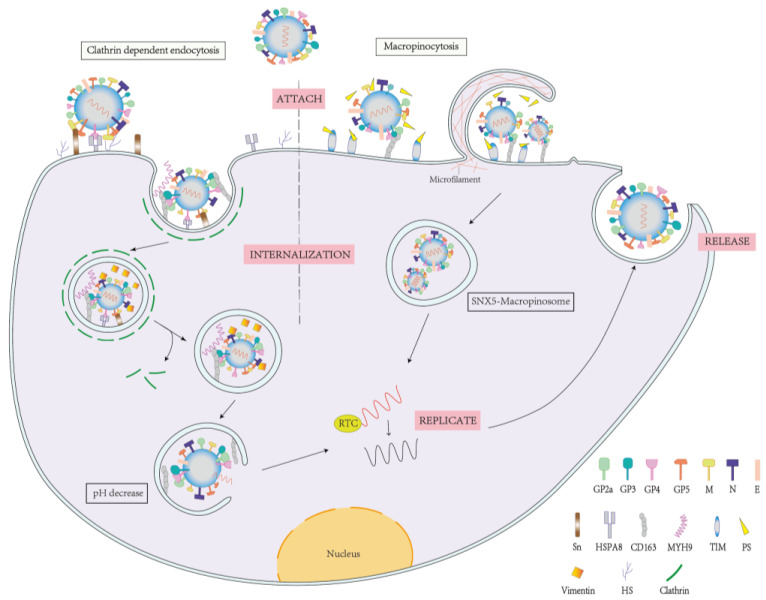
Schematic diagram of PRRSV infection of susceptible cells. The four steps of viral infection in cells are attachment, internalization, replication assembly, and release. This diagram primarily shows how viral membrane proteins and nucleocapsid proteins interact with receptor proteins to enter the cell.

PRRSV primarily enters cells by receptor-mediated endocytosis, and a key step in this process is the binding of several structural and non-structural proteins to the receptor. To boost viral particle adherence and internalization, the glycoprotein 5 (GP5)/membrane protein (M) heterodimer interacts with HS and the N-terminal structural domains of sialoadhesin, which is facilitated by GP4 binding to HSPA8 [17]. In addition to playing the role of a chaperone, HSPA8 also drives clathrin-mediated endocytosis (CME) and decouples lattice proteins from the vesicle membrane [18]. MYH9 serves as a crucial cofactor in this process, connecting with the CD163 scavenger receptor cysteine-rich (SRCR)1-4 structural domain through its C-terminus to facilitate the internalization of PRRSV [19,20]. CD163 interacts with GP2a/GP3/GP4 to allow host cell penetration through low pH-dependent CME [16,21,22]. PRRSV’s cytoplasmic trafficking and replication are aided by the binding of waveform proteins to other cytoskeleton proteins [23,24]. In addition, PRRSV was found to use viral apoptosis mimicry to invade host cells as an alternative route of infection, in which the virus mimics apoptosis by acquiring phosphatidylserine (PS) on the host surface and exposing it to its own vesicle membrane. It uses this viral apoptosis mimicry as a phagocytic signal to enter cells through TIM induction and macropinocytosis involving CD163 [25]. The specific process is shown in Figure 1.

However, there may be some differences in the utilization of receptor molecules by PRRSV-I and PRRSV-II. CD163 is one of the important receptor molecules for PRRSV entry into host cells, and the deletion of the Marc145 CD163 SRCR5 structural domain caused the American type PRRSV virus to lose its infectivity to Marc145 cells [26]. CD163 knockout pigs with complete deletion of the *CD163* gene were not susceptible to either European or American PRRSV, and gene-edited pigs maintained susceptibility to American PRRSV but not to European strains after partial deletion of the *CD163* region (SRCR domain 5 deletion) or replacement of CD163 SRCR domain 5 with human CD163 SRCR domain 8 [27,28], possibly because Lys-58 replacing Glu-58 reduces PRRSV-I infectivity [29].

It can be deduced that PRRSV-I is species-specific for the CD163 receptor given issues of insensitivity to Marc145 cells during the isolation of the PRRSV-I virus and the difficulty of culturing. The issue of the European type PRRSV’s insensitivity to Marc145 during production or in the clinic might be resolved if the PAM cell-derived CD163 (PAM-CD163) molecule is persistently produced in Marc145 cells, which are epithelial cells generated from monkey kidneys. These newly created cell lines are acceptable for European and American type PRRSV culture, as demonstrated by the stable expression of the PAM-CD163 molecule in CRL-2843 cells [30].

The best option for PRRSV-I passaging cultures might be a cell line expressing two or more PRRSV receptor molecules, because some virus strains, such as some NADC30-like and NADC34-like strains of PRRSV-II, are not sensitive to Marc145 cells, while others can adapt to successive passages in the same type of cell. Studies have shown that PRRSV-I is more susceptible to CD163^+^ Sn^+^ cells [31], and that PK15 cells can maintain larger infection titers of PRRSV-I via stable expression of the sialic acid binding Ig, such as lectin 1 (Siglec1) and CD163 receptors [32].

### 2.2. Persistent Infection

Persistent infection is an important feature of Arterivirus, as demonstrated in lactate dehydrogenase elevated virus (LDV) and equine arteritis virus (EAV) infections. Even if the infected pigs are asymptomatic or only exhibit minor symptoms, PRRSV can linger in the body for months or even longer and still cause infection in susceptible pigs when it is regularly, repeatedly, or intermittently discharged into the environment [33]. Acute infection, persistent infection, and disappearance are the three main stages of PRRSV infection. Susceptible pigs are not reinfected when placed in a herd containing infected pigs that were confined for more than 200 days [34,35]. PRRSV can be treated in large, infected herds through confinement management, and it can finally be removed by the immune system of pigs. However, it was hypothesized that PRRSV causes a “lifelong” infection in pigs because of their usual 250-day feeding cycle [13]. Consequently, one of the main causes of the difficulty of PRRSV elimination is prolonged infection.

When pigs become infected, the nascent virus must be assembled, released from the cell, and moved to one or more susceptible cells to continue infecting cells. Intercellular transfer of the virus is subject to multiple inhibitors and effects, including blockage by the immune system, an in vivo environment not conducive to virus survival, and necrosis and apoptosis of infected cells, all of which lead to a progressive reduction in the success of intercellular transfer and eventual elimination of PRRSV [36]. However, it was found that PRRSV was rescued from apoptosis and necrosis by transporting infectious viral RNA, certain replicative enzymes, and certain structural proteins to neighboring cells via intercellular tunneling nanotubes (TNTs), which might be an alternative route for PRRSV transmission between cells [37,38].

The current PRRSV vaccine can only reduce the viral load or duration of infection to treat viremia and slow down clinical lesions after PRRSV infection because of an impaired innate immune response, a weak adaptive immune response, and PRRSV escape mechanisms [39,40,41]. The vaccine cannot completely prevent viral infection, eliminate viremia, produce broad strain protection, or block viral transmission in vivo or in vitro. Additionally, EVA, LDV, and Simian hemorrhagic fever virus (SHFV), which are also arteritis viruses, are selected for lower virulence and immunogenicity during the persistent infection phase, in contrast to stable and highly virulent mutations during the acute infection phase of the virus, which will cause the virus to evolve and generate multiple immune escape mechanisms [42].

## 3. PRRSV and the Adaptive Immune Response

### 3.1. Cellular Immunity

#### 3.1.1. Cellular Immune Response of the Host

Despite having an impact on the thymus and bone marrow, PRRSV infection does not significantly impair lymphocyte differentiation or maturation or cause severe lymphocyte failure or ablation, indicating that the host’s adaptive immune response is not compromised [43,44]. The adaptive immune response includes T cell-mediated cellular immunity (CMI) and humoral immunity (HI), with specific antibody production by effector B cells. Inflammatory responses, dendritic cells, monocytes, and neutrophil marker gene expression levels were shown to be elevated by PRRSV infection using gene set enrichment analysis, while T cells, B cells, and NK cell-related gene markers were downregulated [45].

T cells are essential to the development and control of antigen-specific immune responses, including the induction and activation of B cells, the selection of cytokines and cytotoxic effector functions in the antigen-presenting environment, and the regulation of the immune response [46]. In contrast, B cells are not only crucial to the immune response, but also to immune system maintenance, and they have the capacity to produce cytokines. The activation and growth of particular CD4^+^ cells induced by B and T cells are crucial aspects of the immune response.

According to a recent study, CD69 strongly stimulates CD4^+^ T cells in the lymph and CD8^+^ T cells in the spleen at 14 days after PRRSV infection [47]. Th1 cells, cytotoxic T lymphocytes (CTLs), T-cell receptor-γδT (TCR-γδT) cells, and regulatory T cells (Tregs) show higher polarization in the cellular immune response [48]. Among them, the CTL response is highest in the lungs’ infected regions and in bronchoalveolar lavage [49]. Moreover, CTLs can also improve the placental barrier to PRRSV-1 infection [50]. Helper T cells (Th) are the main responders during viremia and are linked to viremia remission, whereas γδT cells are the main responders following viremia and are essential for the anti-PRRSV response in the lymphatic system [49].

#### 3.1.2. Mechanism of PRRSV in Anti-Cellular Immunity

Through a variety of methods, PRRSV infection can impair healthy thymic function and control immunological responses, lowering or modifying T cell development, delaying and weakening adaptive immune responses, and disrupting cytokine responses [43]. After PRRSV infection of PAMs, it is thought that NK cells in the innate system might also exert adaptive immune response capabilities, dramatically reducing virus vulnerability to NK cell toxicity [51,52].

Previous studies used interferon gamma (IFN-γ) enzyme-linked immunosorbent spot assays (ELISPOT) and immunostaining to detect the cellular response during the infection phase. The results were compared to the local distribution and abundance of PRRSV in the infected tissues, and the infected site’s T cell phenotype was identified. It was discovered that there was a local CMI reaction in both acute and chronic infections, and that γδT cells considerably decreased, particularly in the lungs and lymph nodes. PRRSV impairs T cell identification of infected macrophages, compromising CMI and extending PRRSV infection [53]. In addition, Treg cells are induced to multiply and produce a significant amount of the inhibitory cytokine interleukin 10 (IL-10) in the lungs of infected pigs under any infection state, and interleukin-1 receptor antagonist (IL-1Ra) is involved in immunosuppression, affecting the induced Treg cells in conjunction with IL-10 [54,55]. Furthermore, immunological negative regulatory factors and immune checkpoint molecules (including programmed cell death 1 ligand 1 (PD-L1), PD-L2, IL-10, and transforming growth factor beta 2 (TGFB2)) are elevated as a result of PRRSV infection in a virus-dependent manner [56]. The host’s adaptive immune response is negatively regulated by this overexpression, which also impacts T cell growth, maturation, and selection [57]. One example is the imbalance between co-stimulatory and co-inhibitory immunological checkpoint markers in relation to lung lesions following PRRSV-1 infection [58].

#### 3.1.3. Cellular Immunity and Vaccine Development

Early research suggested that viral matrix peptides might be important for cellular immunity, and several PRRSV proteins, including M, nucleocapsid (N), GP3, GP4, GP5, nonstructural protein (NSP)2, NSP5, and NSP9, have been found to contain T-cell epitopes [59,60]. Meanwhile, researchers have discovered a variety of pertinent peptides, such as those from GP3 and NSP9, by developing T cell-based vaccines using CTL epitopes [61,62,63,64]. They have further identified PRRSV-interacting epitopes from a class 1 major histocompatibility complex (MHC-1) perspective, and they have noted that NSPs are a major source of MHC-I presenting peptides and that the identified epitopes trigger IFN-γ responses in vitro [65].

Currently, researchers are evaluating cellular immune responses or using key factors in the cellular immune response to design novel vaccines that enhance the immune response, as summarized in Table 1.

In conclusion, while PRRSV infection impairs cellular immunity, the immune response’s cellular epitopes can be enhanced through vaccination by combining modified live viruses (MLVs) with an array of vaccine delivery methods. However, the discovery of effective strategies to suppress the escape mechanisms of PRRSV against cellular immunity would be facilitated by further identification of T and B cell epitopes and in-depth research into the processes of PRRSV evasion against NK cells.

### 3.2. Humoral Immunity

Weak neutralizing antibody responses, delayed responses, and the development of significant numbers of non-neutralizing antibodies are the main ways by which PRRSV impacts host humoral immunity [72]. It takes 7–9 days for PRRSV to stimulate the body to produce antibodies; however, these antibodies are not protective and even aggravate the infection, while neutralizing antibodies (Nabs) are not present until 28 days post-infection (dpi) [46].

#### 3.2.1. Delayed Production of Neutralizing Antibodies

Passive transfer of PRRSV Nabs to a sow can prevent gestational reproductive failure and confer immunity to the sow and her offspring. High titers of PRRSV Nabs can protect weaned piglets and reduce viremia and virus transmission, and passive immunization produces neutralizing antibodies for prophylactic protection and homologous protection [73,74]. However, PRRSV infection typically results in a poor and delayed Nab response in pigs. This response is controlled by a variety of parameters, most notably the impact on B cell maturation, non-neutralizing antibody interference, and glycan shielding. Follicular helper T cells (Tfh) signal antibody affinity maturation to B cells and encourage the antiviral effects of IgG2a/c subclasses during infection; however, in the case of PRRSV infection, thymic function is compromised and signal transmission between Tfh and B cells is decreased, delaying the emergence of high-affinity PRRSV Nabs [43,75]. The B-cell antigenic epitopes in the PRRSV structural protein GP5 comprise non-neutralizing epitope A and neutralizing epitope B. At the start of the PRRSV infection, epitope A yields a substantial number of non-neutralizing antibodies while inhibiting the ability of epitope B to trigger the generation of Nabs [76,77]. Furthermore, the extracellular domain of GP5 and the N-glycan of GP3 can prevent the virus from being neutralized by antibodies and reduce the immunogenicity of the neutralizing epitope [78,79,80,81].

#### 3.2.2. Non-Neutralizing Antibodies and ADE

A virus’s capacity to increase the infectivity of immune cells, such as macrophages, monocytes, and granulocytes, and to promote viral proliferation when specific Nabs are at sub-neutralizing levels or when nonspecific antibodies are present, is termed antibody-dependent enhancement (ADE). Immune cell surface receptors, mainly Fc receptors (Fc Rs), complement receptors (CRs), and β2-microglobulin, mediate the effects of ADE caused by viral infection [82,83].

**Figure 2:**
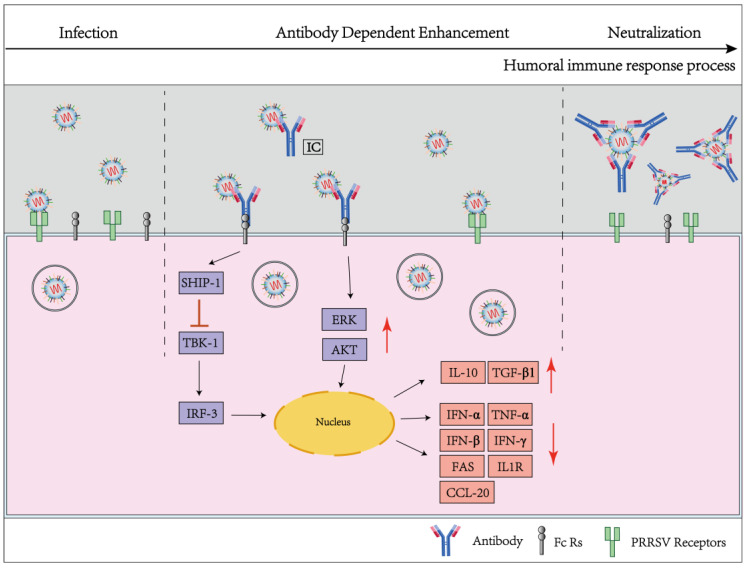
Illustration of the impact of ADE on PRRSV infection. PRRSV particles can be neutralized by high titer specific neutralizing antibodies. Within the influence of ADE, the level of PRRSV infection dramatically increases as compared to the infection stage without antibodies. Additionally, PRRSV binds to non-neutralizing or sub-neutralizing antibodies to create immune complexes (ICs), which enter cells via FcRs receptors to influence innate immune function and to obstruct anti-inflammatory signaling molecules, thereby facilitating virus reproduction.

The body produces antibodies quickly and for several months early in the infection when the antigenic epitopes of PRRSV structural proteins (e.g., GP5, M, and N) and nonstructural proteins (e.g., NSP1, NSP2, NSP4, and NSP7) are present; however, these antibodies are not connected to Nabs. Pigs produce quite a few of these non-neutralizing antibodies with high titers, which bind to the virus to form antibody complexes that mediate virus entrance into cells with the assistance of Fc Rs (primarily FcRI, FcRIIb, FcRIII, and FcεRI), greatly boosting PRRSV infectivity [83,84,85,86] (Figure 2). When PRRSV-ADE affects a cell, it significantly increases the expression of mitochondrial respiratory chain complexes, interferes with antiviral protein function, the ubiquitin–proteasome system, and ribosome function, and changes the intrinsic immune function of PAMs by interfering with innate immune signaling and by impairing the transcription of associated transcription factors [87,88]. The effects of Fc receptor CD16-mediated ADE boost viral infection, suggesting that it might be possible to make PRRSV infect CD16-expressing cells and spread to more organs [89].

However, the relationship between ADE and PRRSV infection in vivo is debatable. Following attenuated vaccination and PRRSV attack, severe sickness after attack has not been shown in immunized animals, and no elevation of infection has been observed [39,74,90]. The link between ADE and PRRSV could be the main focus when it comes to the reproduction of this immunization phenomenon under conditions of more severe clinical illness in pigs [91].

#### 3.2.3. The Virucidal Effect of Non-Neutralizing Antibodies

Non-neutralizing antibodies are generally used to identify or assess whether an animal has been exposed to, or infected with, the virus. As discussed before, PRRSV infection can induce substantial numbers of active non-neutralizing antibodies. Although many of the structural and nonstructural proteins of PRRSV can cause potent humoral immune reactions, the majority of antibodies are unable to neutralize the virus. Recent research has demonstrated that active non-neutralizing antibodies might have a greater effect on the immune response than previously thought. For example, antibody-dependent cell-mediated cytotoxicity (ADCC), antibody-dependent complement-mediated cytotoxicity (CDC), and antibody-dependent cellular phagocytosis (ADCP) all contribute to virus infection or virus clearance in animals. In the process of viral infection, or in the removal of viruses from animals, CDC and ADCP play crucial roles. Studies on the effects of antibodies on pathogens, such as the Ebola virus (EBOV), HIV, and influenza viruses, are currently being conducted. Examples include porcine lgG subclasses that activate CDC, ADCC, and ADCP to protect against H1N1 infection [92], and non-neutralizing or weakly neutralizing antibodies that can neutralize EBOV via ADCP and ADCC [93].

Innate effector cells, primarily NK cells, identify non-neutralizing antibody–viral protein immune complexes on infected cells via FcRs and release cytotoxic factors (including perforin and granzyme) that kill virally infected cells, thus causing ADCC. Through ADCC, NK cells kill infected cells in HIV invasion-susceptible cells and prevent viral replication [94,95]. In addition, genetic variations of the NK cell receptor, FcRIIIa, impact the particular ADCC response to SARS-CoV-2 [96]. Although no studies utilizing ADCC to suppress PRRSV infection have been reported, it is possible that the immune escape mechanism of PRRSV makes innate effector cells, such as NKs, less sensitive, allowing them to evade the ADCC effect. Additional research should be carried out to observe the ADCC response to PRRSV infection.

Through the activation of the complement cascade response, CDC causes inflammation to lyse-infected cells [97]. The ability of PRSSV-specific non-neutralizing active antibodies to wipe out the virus in alveolar macrophages was validated using PRSSV-infected pig alveolar macrophages [98]; however, the precise mechanism of action remains unknown.

Pathogens can also be eliminated by ADCP in addition to ADCC and CDC. In ADCP, FcRs on the surface of macrophages are activated by antibody-conditioned target cells to cause phagocytosis, which internalizes and destroys viral particles. By causing ADCP to swallow viral particles, recombinant cytomegalovirus viral glycoprotein B (gB) vaccination protected against HCMV infection [99,100]. However, ADCP is not currently involved in PRRSV immunological research, which might be related to PRRSV’s predilection for macrophages.

## 4. Conclusions

Porcine reproductive and respiratory syndrome (PRRS) has been a severe veterinary disease of significant economic significance for more than three decades, and it is a highly contagious disease affecting the pig business globally. The current commercial vaccine offers only modest protection for pigs, and specific therapeutic drugs have not yet reached their full potential, making the prevention, control, and eradication of PRRS difficult. The enormous genetic diversity of PRRSV, the induction of persistent infection, the inhibition of the host immune response, and the evasion of innate and adaptive immunity have impeded vaccine development and the development of effective drugs.

Massive replication, shedding, and ongoing transmission of PRRSV in the host are caused by the tardy response of the protective cellular and humoral immunity. Virus removal occurs in the late stages of infection after the production of neutralizing antibodies and cellular immunity to kill virus-infected cells. Therefore, ongoing research on the mechanisms by which PRRSV evades cellular and humoral immunity, the targets of virus–T cell action, the relationship between T cells and the delayed production of neutralizing antibodies, and the protective role of non-neutralizing antibodies could help to clarify these mechanisms and aid in the development of highly protective vaccines.

## Figures and Tables

**Table 1:** Research progress in the design of novel vaccines using cellular immune response properties.

Design System	Ingredients	Test Results	Reference
MnGNP	Mannose-modified gelatin nanoparticles (MnGNP) as a carrier to encapsulate inactivated PRRSV virus	Improves T-cell activation, proliferation, and immunity	[66]
CH/AL-BV	Chitosan/sodium alginate (CH/AL) nanoparticle-encapsulated bee venom (BV)	Effectively induces Th1-related immune responses, stimulates T cells to secrete IFN-γ, and reduces immunosuppressive effects	[67]
VRPs	Expression of PRRSV cytotoxic T cell epitopes using viral replicon particles (VRPs) of swine fever virus	Significantly reduces viral load in the lung tail and improves cell-mediated immune response	[68]
LI-M’	Integration of the hydrophilic structural domain of the PRRSV M protein into Listeria monocytogenes	Significantly enhances CD8^+^ T cell-mediated immunity	[69]
DNA-MLV	DNA vaccines encoding conserved B and T cell epitopes among European subtype 1 strains and known strains	An expanded T-cell response and enhanced antibody response	[70]
IL-15-MLV	Interleukin-15 (IL-15) and MLV fused with glycosylphosphatidylinositol (GPI)	Enhances NK and T cell immune responses and provides some allogenic protection	[71]

## Data Availability

Not applicable.

## Abbreviations

The following abbreviations are used in this manuscript:

GP	Glycoprotein
GP2a	Glycoprotein 2a
GP3	Glycoprotein 3
GP4	Glycoprotein 4
GP5	Glycoprotein 5
M	Membrane protein
N	Nucleocapsid
GP5/M heterodimer	Glycoprotein 5/Membrane protein heterodimer
NSP	Nonstructural protein
NSP2	Nonstructural protein 2
NSP5	Nonstructural protein 5
NSP9	Nonstructural protein 9
PAMs	Pulmonary alveolar macrophages
CD163	Cysteine-rich scavenger receptor
PAM-CD163	PAM cell-derived CD163
Sn or CD169	Sialoadhesin
CD163+ Sn+ cells	exist CD163 and Sn cells
DCs	Dendritic cells
NK	Natural killer
HS	Heparin sulfate
CD151	Cluster of differentiation 151
MYH9	Non-muscle myosin heavy chain 9
HSPA8	Heat shock protein member 8
CME	Clathrin-mediated endocytosis
TIM	T-cell immunoglobulin mucin structural domain
PS	Phosphatidylserine
SRCR	Scavenger receptor cysteine-rich
DC-SIGN or CD209	Dendritic cell-specific intercellular adhesion mole-Cule-3-grabbing non-integrin
Marc145	Epithelial cells generated from monkey kidney
Nabs	Neutralizing antibodies
Tfh	Follicular helper T cells
ADE	Antibody-dependent enhancement
Fc Rs	Immune cell surface receptors, mainly Fc receptors
CRs	Complement receptors
ADCC	Antibody-dependent cell-mediated cytotoxicity
CDC	Antibody-dependent complement-mediated cytotoxicity
ADCP	Antibody-dependent cellular phagocytosis
